# Local Resection in Choroidal Melanoma: A Review

**DOI:** 10.3390/jcm11237156

**Published:** 2022-12-01

**Authors:** Josep Maria Caminal, Daniel Lorenzo, Cristina Gutierrez, Andrea Slocker, Josep Maria Piulats, Estefania Cobos, Pere Garcia-Bru, Rahul Morwani, Juan Francisco Santamaria, Luis Arias

**Affiliations:** 1 Department of Ophthalmology, Ocular Oncology and Vitreoretinal Service, Bellvitge University Hospital, L’Hospitalet de Llobregat, 08907 Barcelona, Spain; 2 Department of Brachytherapy, Catalan Institute of Oncology, Avinguda de la Gran Vía de l’Hospitalet, L’Hospitalet de Llobregat, 08908 Barcelona, Spain; 3 Department of Oncology, Catalan Institute of Oncology, Avinguda de la Gran Vía de l’Hospitalet, L’Hospitalet de Llobregat, 08908 Barcelona, Spain

**Keywords:** uveal melanoma, exoresection, lamellar uveal melanoma resection, scleral resection, transscleral resection, choroidectomy

## Abstract

Surgical resection is widely used to treat small tumours located in the iris and the ciliary body, due to the accessibility of these sites. By contrast, surgical removal of choroidal tumours is substantially more challenging, which is why this procedure is performed only at specialised centres. In the present article, we review the literature on surgical resection of choroidal tumours, which can be performed as endoresection (ab interno) or transscleral resection (ab externo). An important aim of this review is to describe and compare the two approaches in terms of visual outcomes, survival rates, and complications. Both approaches are indicated for the removal of large tumours (thickness > 8 mm) with small base diameters. Surgical resection of the tumour allows clinicians to obtain valuable histopathologic and cytogenetic data from the specimen and eliminates the risks associated with radiotherapy. However, both of these surgical approaches are technically challenging procedures involving the risk of severe early and late postoperative complications.

## 1. Introduction

Multiple options are available for the treatment of uveal melanoma, including several different forms of radiotherapy, transpupillary thermotherapy, enucleation, and surgical excision of the tumour. Consequently, the optimal treatment approach must be individualised according to tumour-related factors (e.g., thickness, base diameter, location, activity, and involvement of surrounding structures) and other factors, such as the patient’s general health status, age, status of the contralateral eye, and the patient’s preferences [1,2].

All of these treatments have advantages and disadvantages, which is why the optimal therapeutic approach remains controversial. In most centres, radiation therapy (brachytherapy and teletherapy) is the first line of treatment (and therefore the most common treatment) in patients with uveal melanoma. Nevertheless, in selected patients, surgical resection may be the treatment of choice, in order to control the intraocular tumour while preserving the eyeball and avoiding the risks of radiation and disfigurement from enucleation. Surgical resection is particularly appealing for the treatment of large uveal melanomas, due to the long-term side effects associated with brachytherapy and teletherapy [2,3].

In cases of uveal melanoma involving iris and ciliary body tumours, local resection is a common treatment. However, surgical resection has been little used in choroidal melanomas, due to the technical difficulty of the procedure and the risk of extrascleral or systemic dissemination. Currently, two distinct surgical techniques are performed. The first is transscleral resection (ab externo technique), also known as “exoresection” and sometimes called iridectomy, cyclectomy, or choroidectomy (and any combination of these), depending on the tumour localisation (iris, ciliary body, or choroid). The second surgical approach is known as endoresection (ab interno technique) (Table 1). Both techniques have advantages and disadvantages, which is why the optimal therapeutic approach is still controversial, particularly for the treatment of large uveal melanomas [4,5,6].

Surgical resection of the tumour may be indicated as a primary or secondary treatment. In certain cases, such as tumour recurrence or toxic tumour syndrome, resection can be a valuable secondary treatment [4,5].

The primary aim of the present article is to review the available scientific literature to describe the main outcomes associated with primary surgical resection of choroidal melanoma (choroidectomy or cyclochoroidectomy). A second aim is to discuss the techniques and indications for the surgical treatment of choroidal melanoma.

## 2. Materials and Methods

We searched the PubMed database of the U.S. Nacional Library of Medicine and the Cochrane Central Register of Controlled Trials of the Cochrane Library using the following keywords: uveal melanoma resection; exoresection; lamellar uveal melanoma resection; scleral resection; transscleral resection; and choroidectomy. The initial search yielded 307 articles.

Next, we applied the following criteria to further refine the selection process for these studies: (1) English language only; (2) reports describing the surgical resection of tumours located in the choroid or the ciliary body and choroid; and (3) reports describing endoresection or exoresection techniques as primary treatments, with or without adjunctive radiotherapy.

After applying these additional criteria, a total of 25 studies remained. Of these 25 articles, 12 involved studies describing exoresection (six retrospective studies and six comparative studies) and 13 were studies describing endoresection (11 retrospective studies and two comparative studies). 

Because our study involved a review of previously published peer-reviewed literature, no institutional approval was required.

## 3. Results

The results of the selected studies are summarised in Table 2, Table 3, Table 4 and Table 5.

## 4. Discussion

### 4.1. TRANSSCLERAL RESECTION (AB EXTERNO)

Transscleral resection is a highly demanding and controversial procedure. It is usually performed under severe controlled systemic hypotension, an approach that entails major risks for the patient. Consequently, it is not surprising that this technique is only performed in a few referral centres. 

Local resection of uveal melanoma was first described in 1911 in a 25-year-old female patient with a ciliary body melanoma [31]. In the 1960s, Stallard described a technique developed to excise choroidal or ciliary body melanomas through a scleral lamellar flap, applying circumferential diathermy at the tumour base and resection of the tumour along with the sclera, a procedure the author called “choroidectomy” [32] However, due to the serious treatment-related complications—loss of vitreous, massive haemorrhage, retinal detachment, and dissemination of tumour cells to the orbit—this technique never gained wide acceptance. 

Innovative approaches to resect larger and more posterior tumours began to emerge in the 1970s, thanks to improvements in microsurgical techniques and instrumentation. Foulds described a technique consisting of scleral lamellar dissection at the base of the tumour, removal of the deep scleral lamella attached to the tumour, and finally microsurgical excision of the tumour followed by closure of the defect through the external scleral lamella [33]. This technique was later modified and improved by other surgeons, which greatly expanded the use of this technique, which became known as partial lamellar sclerouvectomy (PLSU) [8,9,13,34,35,36,37].

Peyman et al. described a procedure [34,38] that was similar to that developed by Meyer-Schwickerath [39]. This technique consisted of en bloc resection of the tumour and the scleral wall after intense photocoagulation and diathermy around the base of the tumour, followed by closure of the defect with a scleral graft. However, this technique never gained widespread acceptance and is no longer performed.

#### 4.1.1. Indications and Contraindications 

In general, transscleral resection is suitable in patients with a tumour where a high rate of complications is expected after radiotherapy, especially conditioned by the height of the tumour. Therefore, the technique is indicated in patients with the following characteristics: good general condition; potential visual possibilities; tumour height from 8 to 10 mm; a relatively small base diameter (<15–16 mm); and anterior localization (ideally nasal quadrants). This technique can also be used as salvage therapy after brachytherapy for eyes with an active tumour or severe exudative retinal detachment, or neovascular glaucoma (toxic tumour syndrome). However, this indication (salvage) is not within the scope of the present article.

Contraindications include any of the following: any systemic condition that does not allow for systemic hypotension or prolonged general anesthesia; a basal diameter >16 mm; and optic nerve involvement.

#### 4.1.2. Surgical Techniques

**Figure 1:**
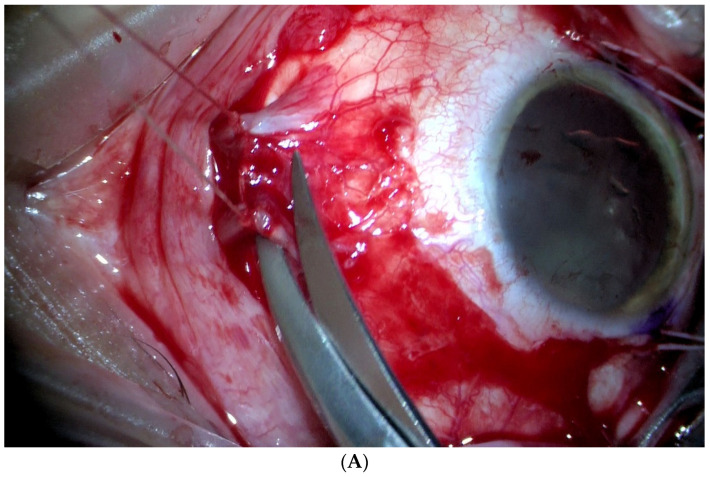

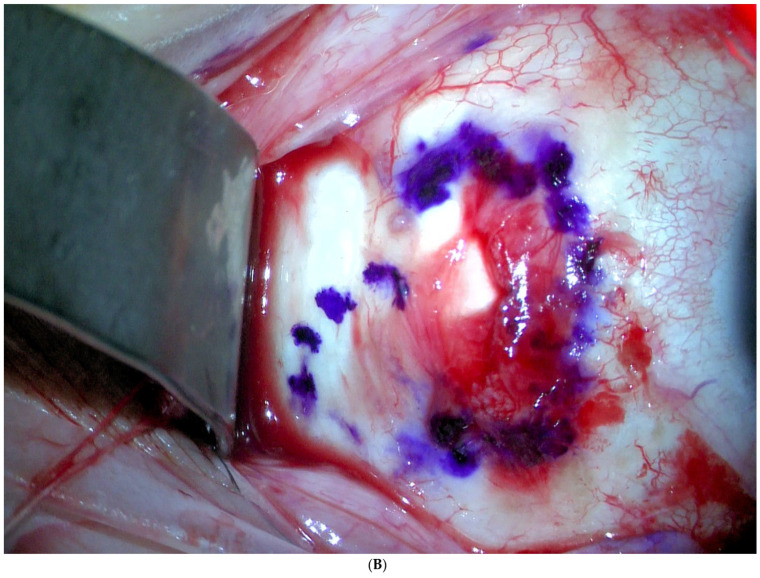

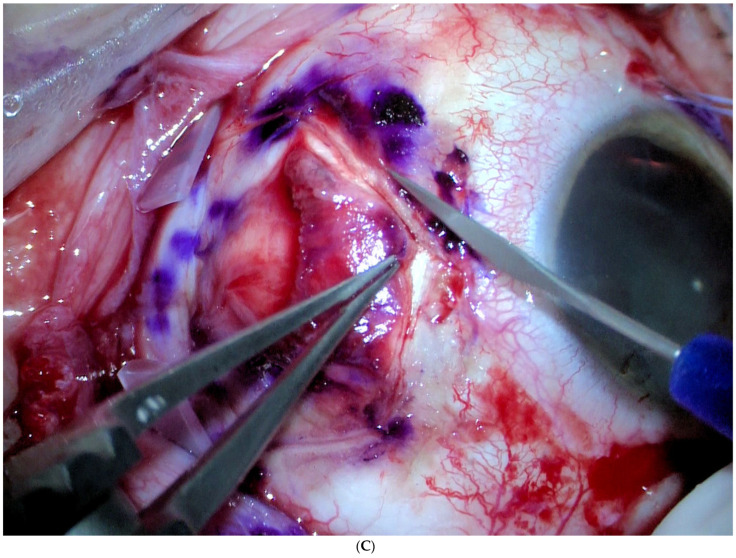

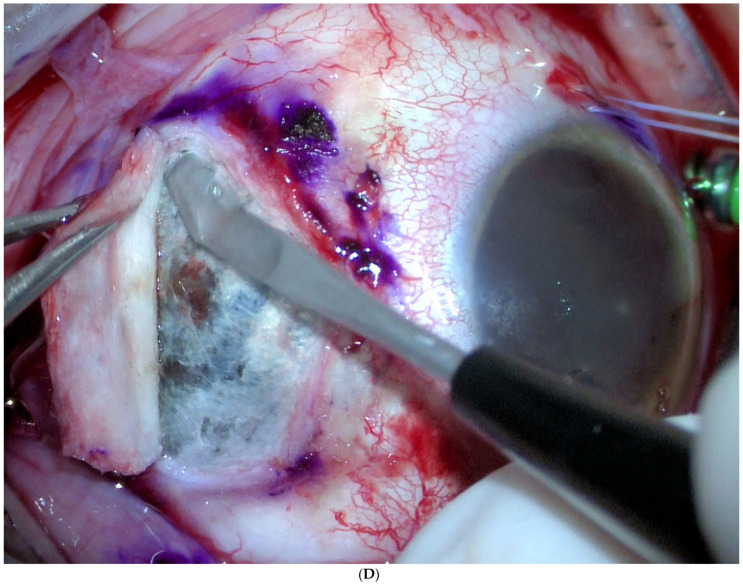

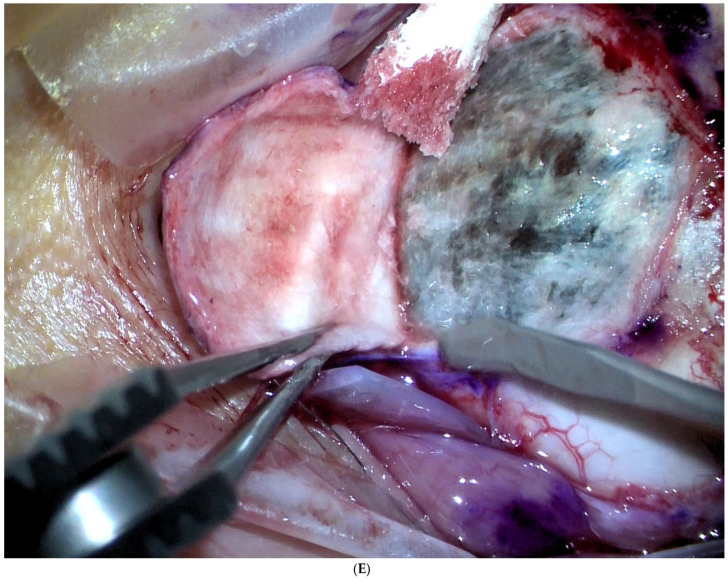

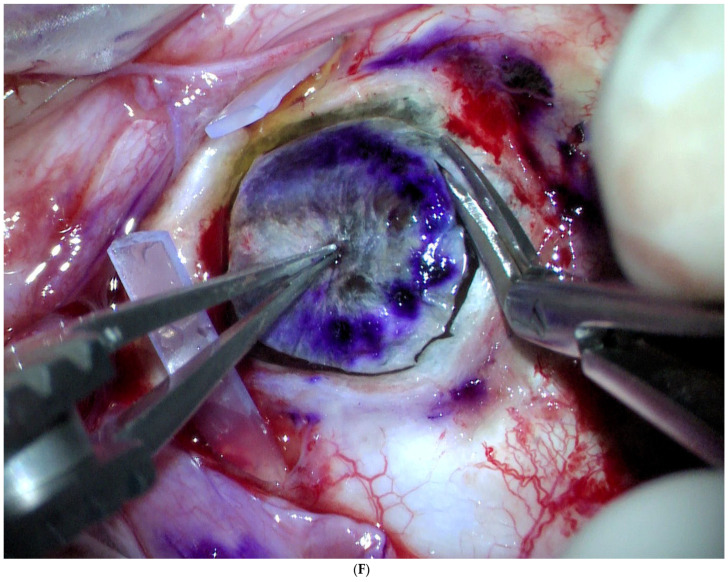

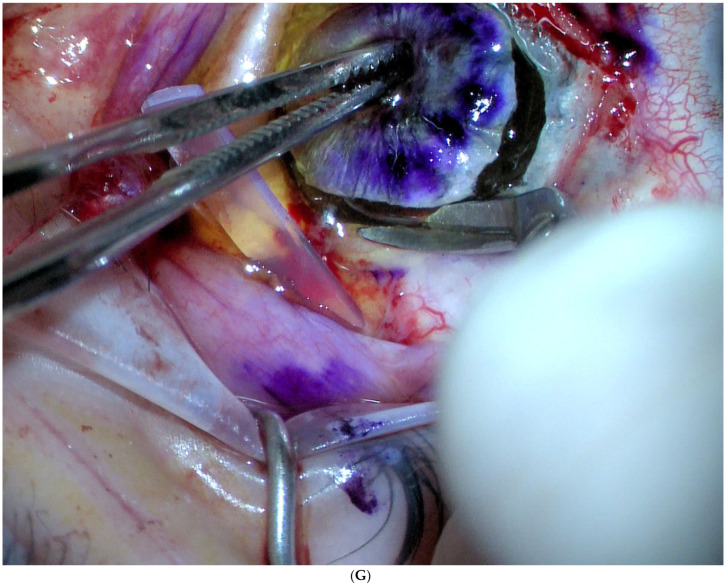

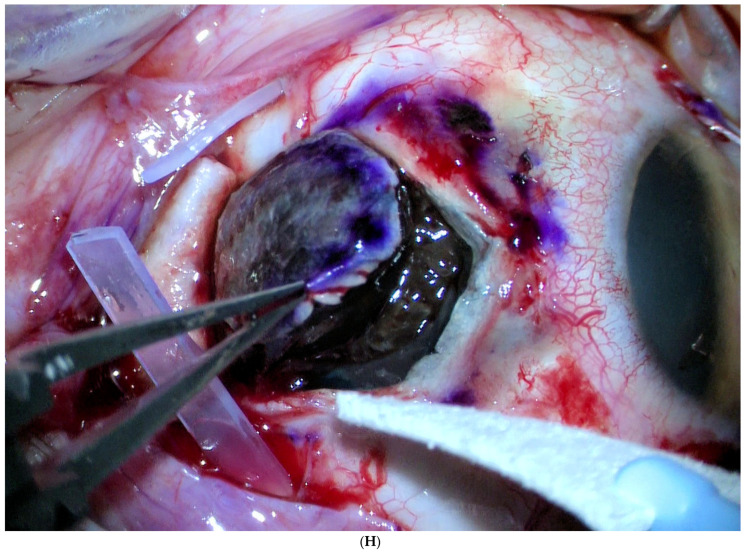

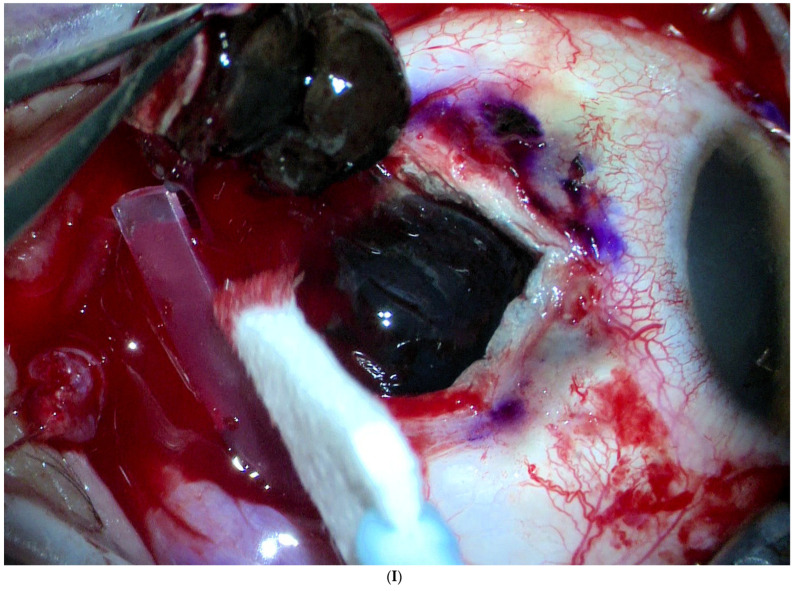

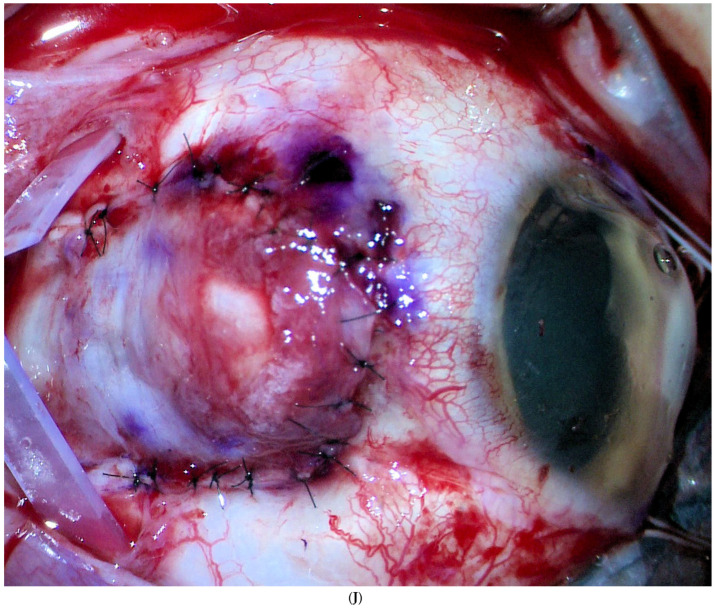
Ab externo scleral resection technique. (**A**). The medial rectus is disinserted to leave the sclera exposed. (**B**). Transillumination and marking of the edges of the tumour base. (**C**). Scleral incision in a polyhedral shape, around the base of the tumour and with 2 mm margins. (**D**,**E**). Scleral flap dissection with a straight scalpel. Further marking of the tumour base by transillumination. (**F**,**G**). Complete section of the inner sclera and exposed uvea around the tumour base with curved and blunt scissors. (**H**,**I**). Careful release of the mushroom-shaped tumour, meticulously separating it from the retina. (**J**). Complete and tight closure of the coloboma with the external scleral flap.

**Figure 2:**
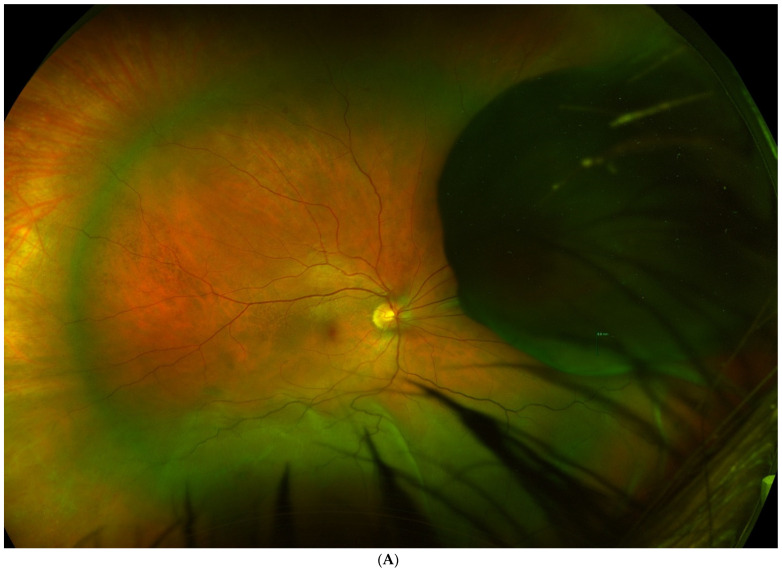

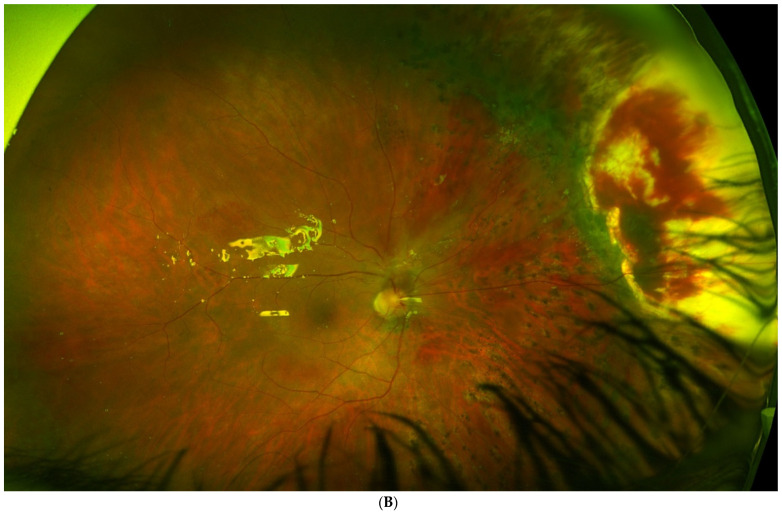
(**A**). Tumour located in the preequatorial and superior nasal quadrant, with extensive inferior retinal detachment, in which a partial lamellar scleral resection was performed. (**B**). Fundus image shows the extensive surgical coloboma, as well as the perilesional photocoagulation and retinal reattachment.

The most common approach to local resection in choroidal or choroidal-ciliary body tumours is PLSU. This technique consists of making a partial thickness circular, rectangular, or polyhedral scleral flap in the area overlying the tumour, after section of the extraocular muscles (if necessary). Next, a scleral incision is made through the deep scleral layers surrounding the melanoma to expose the underlying normal uveal tissue; then, another incision is made to expose the normal retinal tissue. Next, the tumour is removed by en bloc excision along with the deep lamellar scleral flap. At this point, gentle manipulations should be performed in an attempt to remove the tumour together with the overlying inner scleral layer without disturbing the underlying retina or vitreous. Finally, the defect is closed by returning the outer scleral layer to its normal anatomic location and fixing it with multiple interrupted sutures. In most cases, to complete the procedure, adjuvant brachytherapy is performed, which may or may not be associated with complete vitrectomy, to prevent retinal detachment in the case of retinal breaks, as well as lensectomy or phacoemulsification of the lens. During the procedure, systolic blood pressure should be maintained as low as possible (≈40 mmHg), which is why patients at high cardiovascular risk are not considered suitable for this surgical intervention. Nonetheless, it is worth noting that choroidal tumour resections have been successfully performed without systemic hypotension in several studies, with good functional results [4,17,36,37,40,41] (Figure 1 and Figure 2).

#### 4.1.3. Metastasis and Death

Numerous studies have evaluated the risk of metastasis in patients undergoing scleral resection. Unfortunately, most of those studies were small and/or had a short follow-up. Probably the largest (n = 332) and most important study of transscleral resection in this clinical setting was the study by Damato et al [38]. Those authors reported 5-year mortality rates by tumour size (<11 mm, 11–15 mm, and >15 mm of largest basal tumour diameter) of 3%, 20%, and 44%, respectively. On the multivariate analysis, significant predictors of metastatic death were age > 60 years; presence of epithelioid cells; tumour diameter >16 mm; superior localisation; and absence of adjuvant brachytherapy. Metastatic death was not significantly associated with incomplete tumour excision or small residual/recurrent tumours treated by enucleation. In a previous study carried out by the same group of authors [34], the risk of metastatic death was based on the number of risk factors present: (1) basal tumour diameter > 15.4 mm; (2) tumour extension anterior to the ora serrata; and (3) presence of epithelioid cells. In that study, metastatic death at 16 years was 20.2% if none of these risk factors was present, 32.5% with one risk factor, and 46.7% with two risk factors [10,37].

In other studies, mortality rates at 5 and 10 years ranged from 5% to 8.6%, with rates of metastasis from 28% to 44%, respectively (Table 2) [7,8,9,10,12,37]. Data from several non-randomised studies suggested that survival after local resection is not significantly lower than after enucleation or brachytherapy (Table 3) [11,13,14,15,16,17].

#### 4.1.4. Local Tumour Control

Local recurrence may be attributed to incomplete tumour resection or seeding caused by eyeball manipulation during surgery, especially the dissemination of tumour cells, which is promoted by the dispersion of subretinal fluid in the operative field. In most cases, the recurrence develops at the margin, but in rare cases it may be noncontiguous with the primary tumour, thus requiring more aggressive treatment [42]. The presence of a residual tumour is reported in 6% to 13.9% of cases (Table 2) [12,15]. 

Local recurrence rates in choroidal tumours range from 8.3% to 32.6% (Table 1 and Table 2) [15,16] A multivariate analysis carried out by Damato et al. [43] identified several significant predictors of recurrence, as follows: epithelioid cellularity; posterior tumour extension to <1 disc diameter (DD) of the disc or fovea; large tumour diameter ≥ 16 mm; and lack of adjunctive plaque radiotherapy. In that study, the recurrence rate at 4 years was 6% if none of these risk factors were present and 57% if more than two risk factors were present [43].

In another study by Damato et al. [37], 8-year actuarial rates of local tumour control were 75.2% in the absence of any risk factors for recurrence, 71.2% with one risk factor, and 47.1% with more than one risk factor. The risk factors for local recurrence were as follows: (1) basal tumour diameter > 15.4 mm; (2) posterior tumour extension to within 3.0 mm of the optic disk or fovea; and (3) the presence of epithelioid cells. 

Bechrakis et al. [12] reported 5- and 10-year actuarial recurrence rates between 24% and 32%. Several variables significantly impacted these rates, including older age, a large basal tumour diameter, lack of adjuvant ruthenium brachytherapy, and retinal detachment. Puusaari et al. [11] reported a 5-year actuarial local recurrence rate of 41%, which ranged from 37% with adjunct brachytherapy to 46% without it. 

Caminal et al. [17] reported a 5-year actuarial local recurrence rate of 17.5%. Notably, all of the recurrences occurred in patients who did not receive adjunctive brachytherapy. Other studies showed that adjunctive brachytherapy significantly reduces local recurrence, although it also increases the risk of important complications, such as wound dehiscence, maculopathy, and optic neuropathy [43,44] Extraocular recurrence is relatively rare, affecting from 2% to 4% of patients [13,16].

#### 4.1.5. Enucleation 

Enucleation rates range from 11.1% to 32.3%, depending on the study (Table 3). [7,15] In the study by Damato et al. [37], the 8-year actuarial eye conservation rate was 57.1% in patients with ≥one ocular risk factor for adverse outcomes versus 81% in cases with no risk factors. In cases in which complete tumour resection was achieved without retinal tearing, nearly 90% of eyes were conserved at 8 years, regardless of the number of risk factors. Those authors categorised the risk of adverse ocular outcomes according to the number of features present, as follows: (1) basal diameter > 15.4 mm, (2) thickness > 8.4 mm, and (3) posterior extension to within 3.0 mm of the optic disk or fovea. 

In the studies by Puusaari et al. [11] and Caminal et al. [17], the 5-year actuarial enucleation rates were 28% and 29.1%, respectively.

#### 4.1.6. Complications and Visual Outcomes

This type of surgery is associated with the risk of many different complications, both early and late. The most common early complications are vitreous hemorrhage, hyphema, subretinal haemorrhage, retinal detachment, corneal edema, and glaucoma. Late complications include cataract formation, subretinal fibrosis, posterior synechiae, retinal detachment with or without vitreoretinal proliferation, cystoid macular edema, glaucoma, scleral thinning and, very rarely, iris neovascularization. Overall, the most common complications are retinal haemorrhage, retinal detachment, preretinal or subretinal fibrosis, and cataract (Table 3) [7,9,37,45].

In a study of 156 patients, Damato et al. [45] found that 18% developed retinal detachment, most commonly in the early postoperative period, although the detachment was resolved with early vitreoretinal surgery in most cases (84%). According to those authors, retinal detachment was more common in eyes with large tumours and in males; posterior tumour extension was a possible risk factor. The 2-year complication rate for retinal detachment in the actuarial analysis was 16.6% in tumours <6 mm in height and 34% in tumours >9 mm in thickness [37].

Foulds et al. [8] compared local resection to enucleation, finding that 60% of patients who underwent resection retained useful vision and 25% of patients had good vision. In the study by Shields et al. [9], who evaluated PLSU, postoperative VA was equal to or better than preoperative visual acuity (VA) in 24% of cases.

In another study by Damato et al. [46], the multivariate analysis showed that the most significant preoperative predictors of retaining good vision (≥6/12) were nasal tumour location and distance >1 DD between the tumour and the optic disc or fovea. In addition, 57% of patients with a nasal melanoma, without posterior extension, maintained VA >1/10, with 93% maintaining a vision of counting fingers or better. By contrast, none of the patients with a temporal tumour and posterior extension retained vision of 6/12 or better; 50% had vision of counting fingers or better. 

Damato et al. [37] reported 8-year actuarial rates of conservation of vision of counting fingers or better according to the number of risk factors present: 63.9% if no risk factors were present, 60.1% with one risk factor, and 43.5% with ≥one risk factor. The risk of adverse ocular outcomes was determined by the number of features present: (1) basal diameter > 15.4 mm; (2) thickness > 8.4 mm; and (3) posterior extension to within 3.0 mm of the optic disk or fovea. In the 148 patients with an initial VA of 20/40 or better, the 8-year actuarial conservation rate of 20/40 VA or better was 42.8% in the absence of any ocular risk factors and 37.7% if one risk factor was present. 

#### 4.1.7. Comparison between Treatments: Enucleation and Brachytherapy (Table 3)

Head-to-head trials comparing exoresection to other standard treatments for uveal melanoma are scarce. One retrospective study compared transscleral resection to enucleation [13] and five other retrospective studies compared exoresection to brachytherapy using matched pairing [11,14,15,16,17]. 

In the study by Foulds et al. [13] the 5-year overall survival (OS) rate in patients with choroidal melanoma who underwent local resection was 79% (18% tumour-related mortality) versus 54% (47% tumour-related mortality) in patients who underwent enucleation. In both treatment modalities, survival outcomes were substantially influenced by the preoperative tumour diameter: for tumours ≤15 mm in diameter, the 5-year mortality rates were 11.6% for resection versus 30% for enucleation; by contrast, for larger tumours (>16 mm), the mortality rates were 57% and 65%, respectively. Based on those findings, the authors concluded that local surgical resection can achieve a cosmetically satisfactory globe in about 80% of cases and useful vision in about two-thirds of cases. For choroidal tumours up to 15 mm in diameter, particularly if they do not involve the ciliary body, local surgical resection appears to be a more appealing treatment option than enucleation, especially because it does not appear to be associated with any increase in mortality. 

Augsburger et al. [14] compared local resection to cobalt-60 brachytherapy. All patients were matched in terms of age, tumour size (largest dimension), and the location of the anterior and posterior margins. They found no significant differences in mortality between the two groups, with actuarial 5-year survival rates of 85.2% in the resection group and 81.8% in the brachytherapy group. However, the rate of severe early vision loss was greater in the resection group. Notwithstanding the findings of that study, two other studies—by Kivela et al. [16] and Bechrakis et al. [15]—found that transscleral resection yielded better visual outcomes than iodine-125 brachytherapy, with comparable disease-free survival and enucleation rates. Kivela et al. compared transscleral resection to iodine brachytherapy in patients diagnosed with choroidal melanoma ≥6 mm in thickness. The complication rate (macular edema, vitreous hemorrhage, and cataract) was higher in the brachytherapy group. Importantly, rubeosis iridis, neovascular glaucoma, and optic neuropathy occurred only in the brachytherapy group. By contrast, the risk of local tumour recurrence was eight times higher in the surgical resection group, although this had no impact on 8-year survival rates in that group. Bechrakis et al. [15] found that the risk of developing neovascular glaucoma was significantly greater in the brachytherapy group compared to transscleral resection (33.3% vs. 5.6%). No differences between the groups were found in terms of eye retention and mortality rates.

Puusaari et al. [11], who compared transscleral local resection to iodine brachytherapy, found that transscleral resection was associated with a higher risk of local recurrence: at 5 years, the actuarial recurrence rate was 41% in the resection group vs. 7% in the brachytherapy group. Nevertheless, patients who underwent resection were more likely to preserve VA >20/400: the cumulative incidence of loss of 20/400 VA at one, two, and three years in the resection group was 53%, 60%, and 60%, respectively, versus 60%, 75%, and 91% in the brachytherapy group. No between-group differences were observed with regard to eye retention and mortality rates.

Caminal et al. [17] compared transscleral resection without controlled hypotension to brachytherapy, finding that resection resulted in significantly better vision than brachytherapy, with no significant between-group differences in survival, local recurrence, or enucleation rates. Importantly, these outcomes were achieved without the limitations inherent to hypotensive anaesthesia. The most common complications in the brachytherapy group were radiation-induced retinopathy (45.3%), neovascular glaucoma (28.3%), and macular oedema (24.5%) versus rhegmatogenous retinal detachment (21.1%), ocular hypertension (21.1%), and submacular haemorrhage (15.8%) in the scleral resection group.

#### 4.1.8. Conclusions

Transscleral resection was developed due to the need to achieve better visual outcomes than those provided by brachytherapy, without compromising local control or survival. Developments in vitreoretinal surgical techniques have made it possible to avoid hypotension, which is potentially dangerous for the patient; in addition, they can help to prevent the two main complications (local recurrence and rhegmatogenous retinal detachment) associated with this surgical approach. Moreover, the functional outcomes obtained with this surgical technique have substantially improved, as have the number of indications for this surgery, thus increasing the number of eligible patients. 

In conclusion, this surgical procedure has a high technical complexity. Candidates for this procedure include patients in good general condition with the potential to maintain vision with large tumours (8 to 10 mm in height), with a relatively small basal diameter (<15–16 mm), and an anterior location (preferably in nasal quadrants). This procedure is particularly valuable in patients with the aforementioned characteristics, due to the high complication rate associated with brachytherapy, including cataracts, neovascular glaucoma, and radiation retinopathy. Ideally, this intervention should only be performed by highly experienced surgeons in patients who are highly motivated to preserve their eyes and their vision.

### 4.2. ENDORESECTION (OR RESECTION AB INTERNO)

The endoresection approach to the treatment of uveal melanoma is controversial, due to the technical complexity of the procedure and concerns about the risk of intraocular, extraocular, and, most importantly, systemic spread. During endoresection, the tumour is fragmented into small pieces, which goes against the basic principle of cancer treatment of maintaining tumour integrity. Consequently, this technique is performed in only a few specialised referral centres.

Peyman et al. were the first to describe this approach, which involves tumour resection through an internal approach and vitrectomy [47,48]. Those authors named this approach ab interno retinochoroidectomy (or ab interno resection). In a subsequent study, Damato et al. [19] treated 52 patients with this same approach, which they called “endoresection”. Later, García-Arumí et al. [20] described favourable clinical outcomes in a series of patients with large tumour height and a small base who were treated with this technique. Despite these promising results, Robertson [49] raised several concerns about this technique. The first concern was related to the risk of incomplete resection, as it is often impossible to evaluate the margins in the choroid or the presence of retinal invasion. A second concern was the risk of residual intrascleral melanoma, despite laser treatment of the base (extrascleral extension could be associated with residual tumour in the scleral canals). A third risk was the potential to cause the irretrievable dissemination and implantation of malignant cells in the vitreous cavity. A fourth concern was that this approach violated the generally accepted principle of surgical resection of tumours, which posits that transection or incomplete removal of a tumour should be avoided whenever possible. By violating this historic principle, the likelihood of achieving a cure is reduced. Nevertheless, these initial concerns have been allayed through the growing body of evidence from numerous large studies with long follow-up [23,24,25,26,27,28,30]. Moreover, Caminal et al. [29] compared primary endoresection to primary I-125 brachytherapy, finding no statistically significant differences in OS, disease-specific survival (DSS), VA, local recurrence, or eye retention between the two procedures. 

#### 4.2.1. Indications and Contraindications

In general, this technique is indicated in patients with tumours that are not amenable to brachytherapy or in whom vision is unlikely to be preserved. This technique is appropriate for patients in good general condition, with large tumours (8–10 mm in height) with a relatively small basal diameter (<15–16 mm), and a posterior location (juxtapapillary), ideally in nasal quadrants. This technique has also been considered as salvage therapy after brachytherapy for eyes that still show an active tumour, and in eyes with exudative retinal detachment or neovascular glaucoma (toxic tumour syndrome). However, these latter indications are beyond the scope of this article.

Contraindications for this technique include any of the following: systemic conditions that prevent prolonged general anesthesia; basal diameter >16 mm; involvement of the ciliary body; and extensive involvement of the optic nerve.

#### 4.2.2. Surgical Technique

The most widely used endoresection technique is vitrectomy via conventional 20–23 G pars plana, lensectomy or phacoemulsification of the lens, induction of vitreous detachment, perilesional laser endophotocoagulation, or transretinal tumour resection through the vitrectome if the retina is invaded; otherwise, the technique is carried out by means of a peripheral retinectomy, in which the retina is folded away from the tumour, when the retina is not invaded.

**Figure 3:**
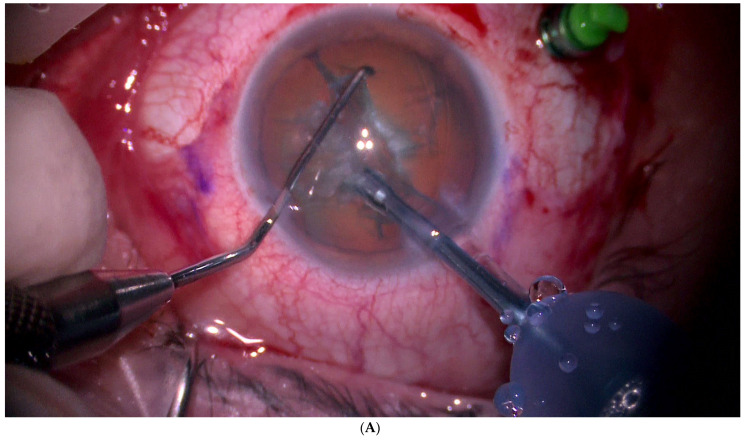

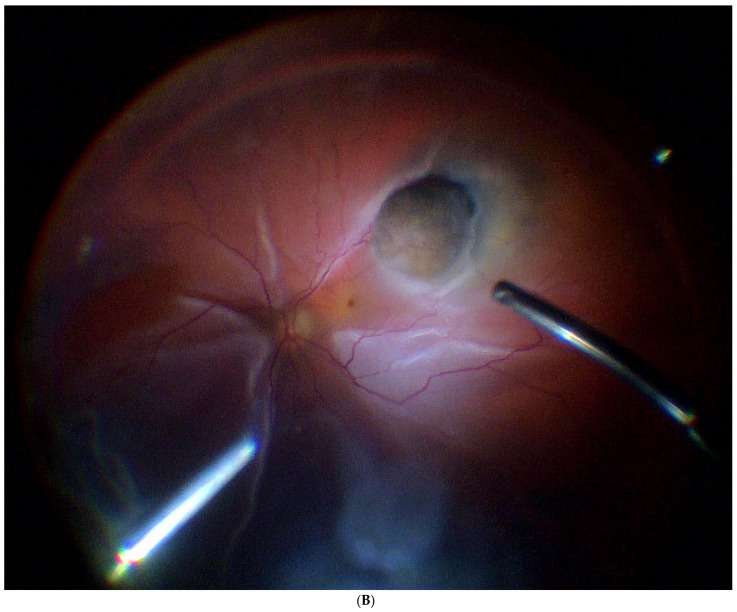

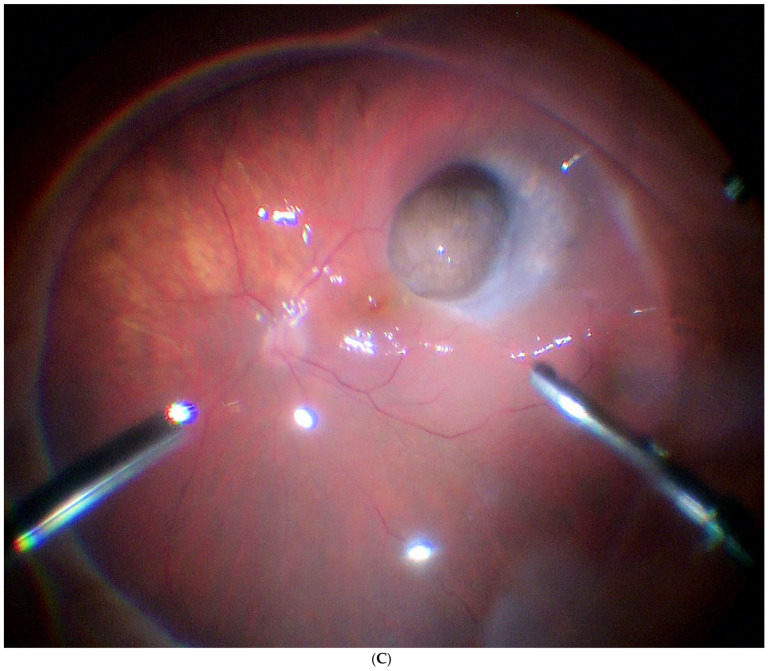

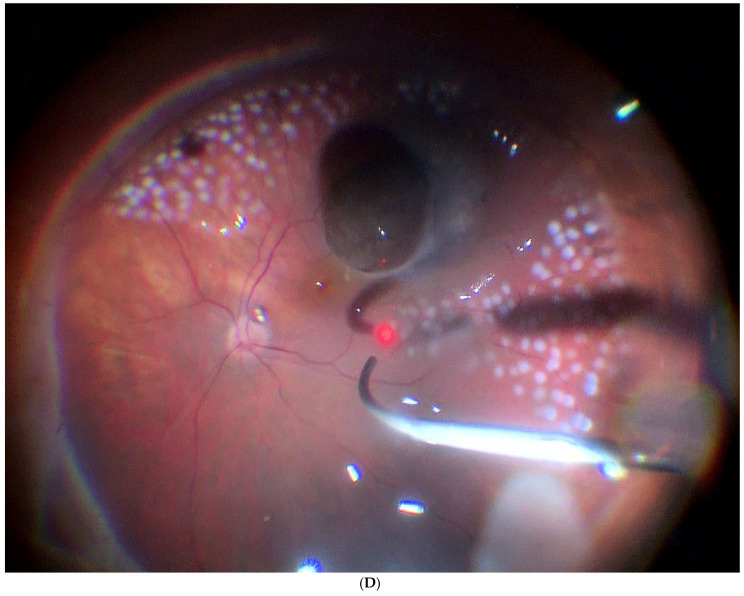

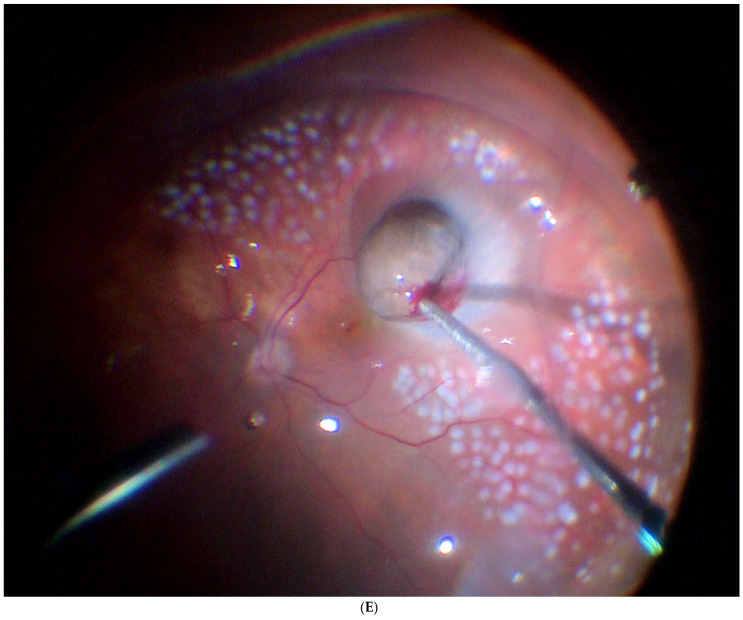

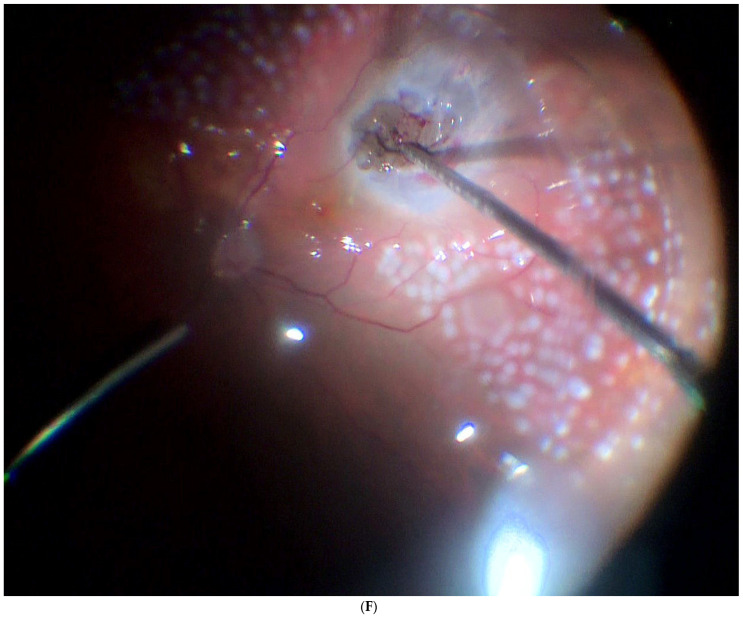

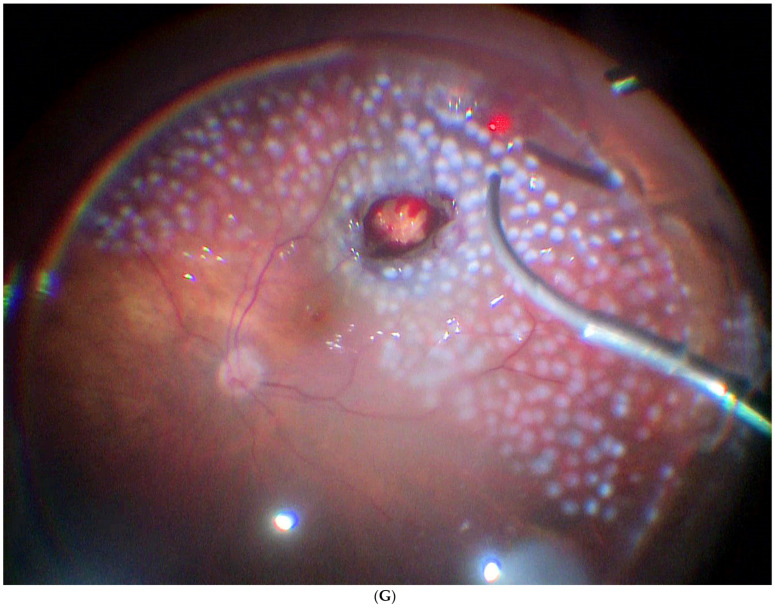

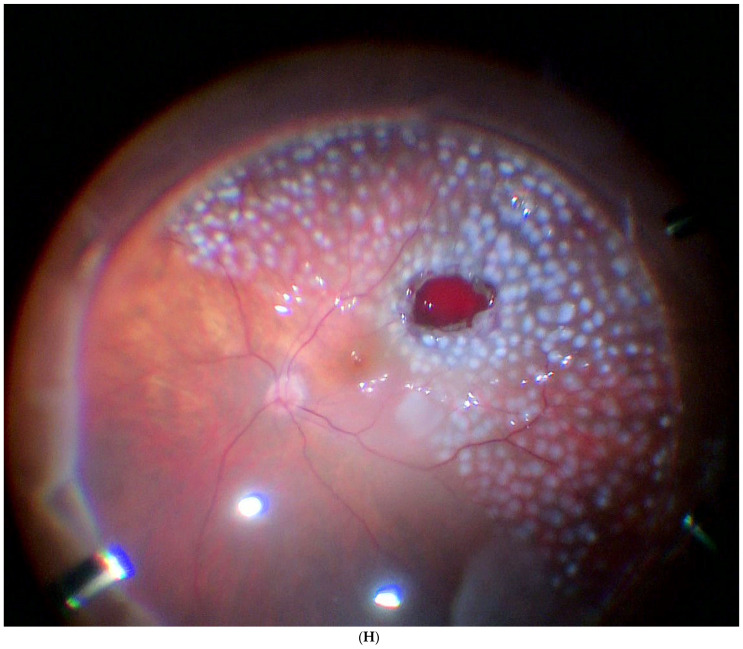
Ab interno resection technique. (**A**). First, phacoemulsification cataract surgery is performed. (**B**). Mushroom-shaped choroidal melanoma located on the superior temporal arcade with extensive retinal detachment. (**C**). Liquid perfuorocarbon is injected to flatten the retina. (**D**). Endophotocoagulation is performed around the tumour. (**E**). Resection is initiated by introducing the vitrectomy probe into the tumour. A low cutting rate and high aspiration is programmed, and the tumour is fragmented into pieces and aspirated. (**F**,**G**). The resection is completed until the bare sclera is reached. It is possible to leave a remnant since it will be treated by brachytherapy. (**H**). Perfluorocarbon liquid is then exchanged for silicone. At this stage, a hemorrhage may occur, which overlies the coloboma. Finally, a radioactive plaque is placed at the base of the tumour.

**Figure 4:**
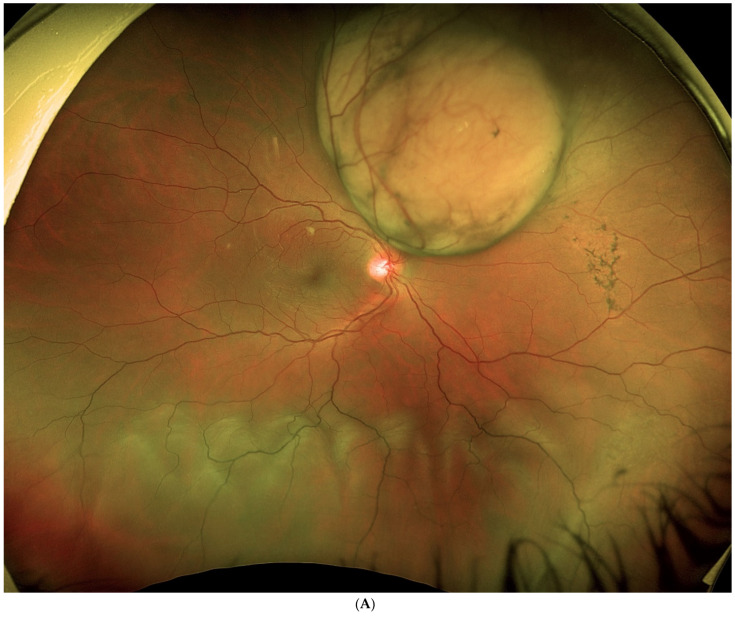

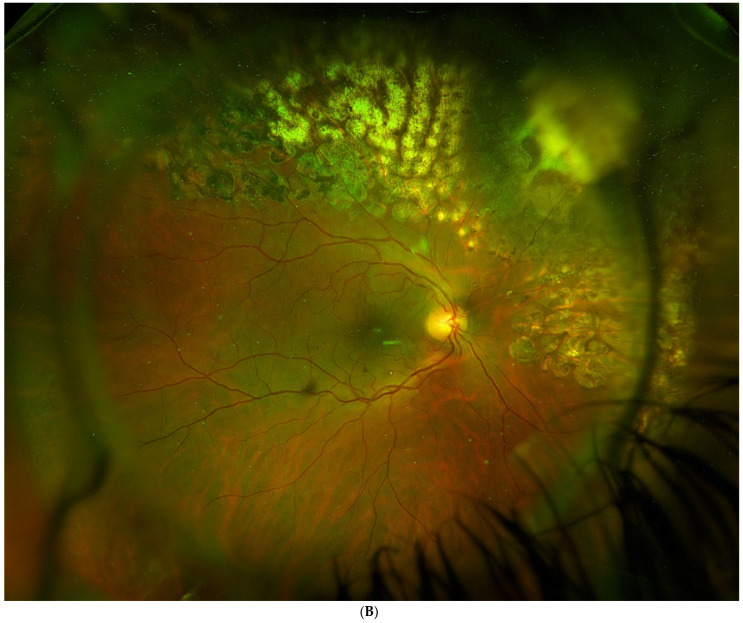
(**A**). Tumour located superior and postequatorial, with extensive inferior retinal detachment, in which an endoresection was performed. (**B**). Fundus image shows the surgical coloboma, as well as the perilesional photocoagulation and retinal reattachment.

A vitreous cutter is used to remove the tumour piecemeal; to control intraoperative bleeding during the procedure, intraocular pressure is raised significantly. The tumour is resected up to the sclera and the retina is flattened by injecting liquid perfluorocarbon. Endolaser treatment is administered to the choroidal edges of the resection and to the exposed scleral surface; photocoagulation is used to reduce bleeding and to destroy any remnants. Thereafter, direct liquid perfluorocarbon–silicone is performed. Finally, an episcleral radioactive plaque is placed at the base of the tumour to treat any tumour remnants and cryotherapy is performed over the sclerotomies. A wide-field lens and scleral indentation allow for complete removal of the tumour by the vitrectomy probe of high equatorial melanomas up to the ciliary body [50] (Figure 3 and Figure 4).

When this technique was first performed, an air–silicone exchange was used to flatten the retina after the tumour had been removed; however, this maneuver can cause a potentially lethal syndrome known as presumed air by vitrectomy embolisation (PAVE). Therefore, it is strongly advised to avoid this maneuver and instead perform a direct exchange of perfluorocarbon liquid with silicone [51,52,53,54,55].

#### 4.2.3. Metastasis and Death

Although endoresection has been widely questioned because it does not follow the classic concept of en bloc tumour removal with safety margins, it is currently widely used because studies have demonstrated good long-term results with this approach. Initially, there were some concerns regarding the risk of incomplete resection, implantation of cancer cells in the vitreous, and dissemination of tumour cells into the bloodstream (due to piecemeal removal of the mass with the vitreous cutter), as any of these could theoretically increase systemic disease or local recurrence. However, the available data have shown that these risks are lower than originally believed. For example, Suesskind et al. [56] found no difference in circulating melanoma cells in peripheral blood after primary enucleation, stereotactic radiotherapy, brachytherapy, transpupillary therapy, or endoresection.

Retrospective case series show cancer-specific mortality rates ranging from 0% to 20%, although follow-up outcomes are highly variable [18,19,20,22,23,24,25,26,27,28,29,30] (Table 3). Actuarial (Kaplan–Meier) survival rates at 5 years range from 90.9% (39) to 100% (46); at 10 years, survival rates range from 57.9% [28] to 97.6% [25] (Table 4). These data are similar to those obtained in the COMS study, in which 5-year overall and disease-specific mortality rates for medium-sized melanomas were 20% and 10%, respectively [57].

Only two studies, both retrospective, have compared primary endoresection to primary brachytherapy for the treatment of melanoma. Neither of those studies found significant differences in OS or disease-specific survival [29,30]. Caminal et al. [29] compared primary endoresection to primary I-125 brachytherapy. In that study, cases were matched according to tumour height and postequatorial tumour location. No statistically significant differences between the two groups were observed in OS, metastasis-free survival, VA, or eye retention (Table 5).

#### 4.2.4. Local Tumour Control

Reported rates of long-term local recurrence and enucleation are quite variable, ranging from 0% to as high as 23% [20,28]. Susskind et al. [28] reported 5- and 10-year actuarial rates of recurrence of 22.7% and 29.2%, respectively (Table 4). Caminal et al. [29] found a recurrence rate of 12.2% at 5 years. Rice et al. [30] compared brachytherapy to endoresection, with similar recurrence rates (Table 5).

The use of adjuvant brachytherapy is associated with a lower rate of local recurrence [25,29]. In fact, it is now generally accepted that adjuvant radiation after tumour resection can reduce the incidence of recurrences, as well as the incidence of metastases [10,11,15,16,43]. Recurrences at the intrascleral and extraocular-orbital level have also been described in isolated cases [58,59].

#### 4.2.5. Complications and Functional Outcomes

The main surgical complications, from most to least common, are scleral bed haemorrhage, cataracts, ocular hypertension, retinal detachment, hemovitreous, macular traction, epiretinal membranes, and bullous keratopathy followed by retinopathy and optic nerve atrophy, both of which are associated with adjuvant radiotherapy. However, the incidence of these complications varies substantially depending on the series, with some authors reporting cataracts in up to 90% of cases, retinal detachment in 33%, ocular hypertension in 34%, and vitreous haemorrhage in 80%. (Table 4) [18,19,20,22,23,24,25,26,27].

Recently, a pulmonary embolism syndrome associated with endoresection (presumed air vitrectomy embolisation (PAVE)) associated with air exchange has been described [52,53,54,55]. Air embolism is a rare complication of ophthalmic surgery that can occur when the choroidal vasculature is exposed to infused air, which can then enter the bloodstream, leading to important systemic effects, such as pulmonary or cardiac embolism. Endoresection involves the removal of all choroidal tissue in the region of the tumour, ideally resecting tissue down up to bare sclera. Through ampulliform dilatation of the vortex veins, the vascular stream is exposed to air embolism. Therefore, it is imperative to avoid infusing air in these patients; instead, direct exchange from liquid perfluorocarbon to silicone should be used. As a preventive measure, ligation of the vortex vein(s) by draining the excision site has been suggested, but vortex vein ligation may lead to acute choroidal haemorrhage [51,52,53,54,55,60,61].

In terms of functional outcomes, most published series have found that 20% to 30% of patients achieve a final vision above 20/200 and a mean vision between 20/40 and 20/100 [18,19,20,21,22,23,24,25,26,27]. Kertes et al. [18] found that 31.2% of patients had a VA of 6/60 or better, with 56.3% between 6/120 and light perception and 12.5% with no light perception. In a study by Konstantinidis et al. [24], VA was better than 6/12 in 13% of cases and better than 6/30 in 31% of cases. In a study by Biewald et al. [26], 13.4% of patients had a final VA of 20/50 or better, while 33.6% had a final VA between 20/400 and 20/50. (Table 4 and Table 5)

#### 4.2.6. Comparison between Treatments: Brachytherapy (Table 4)

Head-to-head trials comparing exoresection to other standard treatments for uveal melanoma are scant. In fact, there are only two studies, both retrospective, that have compared endoresection to brachytherapy [29,30] (Table 5) Caminal et al. [29] compared primary endoresection to primary I-125 brachytherapy, finding no statistically significant between-group differences in OS, DSS, VA, local recurrence, or eye retention. In that study, patients were matched according to tumour height and location. On the Kaplan–Meier analysis, 100% of patients in the endoresection group were metastasis free at 5 years versus 84.2% in the brachytherapy group. At 5 years, OS was 89.2% in the endoresection group vs. 81.5% in the brachytherapy group, respectively, while the DSS (melanoma-related) was 100% versus 84.2%, respectively. In the endoresection group, 92.4% of patients were free of local recurrence versus 96.6% in the brachytherapy group. At 5 years, 87.8% of patients in the endoresection group conserved the eyeball versus 85.7% in the brachytherapy group. There were no differences between groups in final VA. 

Rice et al. [30] compared endoresection to I-125 brachytherapy, finding no significant differences (actuarial analysis) in time to enucleation or time to metastasis or death from any cause. However, it is worth noting that the endoresection group (n = 22) consisted of patients treated at different centres (four different surgeons). In any case, the likelihood of achieving a VA of 3/60 or better was 22% higher in the endoresection group after adjusting for pre-treatment VA, tumour height and diameter, distance from the disc and fovea, and the presence of exudative retinal detachment. The outcomes observed in this small cohort suggest that endoresection may achieve better visual outcomes than brachytherapy in selected cases. 

#### 4.2.7. Conclusions

In conclusion, endoresection is a highly complex surgical procedure. Despite initial concerns about the risks of local or metastatic spread, the available data show that endoresection achieves survival rates that are comparable to those obtained with brachytherapy. Nonetheless, the technical complexity of this procedure limits its use to a small number of specialised centres, mainly in cases in which primary radiotherapy is considered unsuitable. 

Over the past two decades, major advances have been made in ocular microsurgery, especially with the introduction of wide-field systems and new vitreoretinal devices, which have helped to improve surgical outcomes in these patients, especially visual function.

Endoresection may be indicated in patients who are in good general condition and who may be able to preserve their vision, with large tumours (>8 mm in height) with a relatively small basal diameter (<15–16 mm), a postequatorial location, and preferably in the nasal quadrants. In these cases, high complication rates are expected with brachytherapy, particularly retinopathy, radiation neuropathy, and neovascular glaucoma.

It is now generally accepted that adjuvant radiation after tumour resection can reduce the incidence of both recurrences and metastases [10,11,15,16,43].

## 5. Conclusions

KEY POINTS:

- Choroidal tumour resection is an alternative to brachytherapy in selected cases. The available data suggest that this approach may offer better visual results and eye sparing without compromising local tumour control and survival.
- Resection techniques for choroidal tumours are based on modern vitreoretinal surgical techniques, which allow for the treatment of more challenging cases.
- The main limitations to surgical resection of these tumours are the risk of severe complications, which may include retinal detachment, proliferative vitreoretinopathy, severe intraoperative haemorrhage, air embolism, and local tumour recurrence at the resection edge.
- Surgical resection of uveal melanomas should be limited to centres with substantial experience in the management of intraocular tumours.

## Figures and Tables

**Table 1:** Types of local resection.

Local Resection of Uveal Melanoma *
Exoresection (ab externo technique) Iridectomy Cyclectomy Choroidectomy Any combination of the terms above Endoresection (ab interno technique)

* Can be combined with adjunct radiotherapy (brachytherapy, proton beam, or stereotactic radiotherapy).

**Table 2:** Summary of descriptive case series of partial lamellar sclerouvectomy for uveal melanoma.

Author	Patients, n	Diameter, mm	Thickness, mm	Follow-Up, mean	Mortality/Survival	Enucleation	Recurrence	VA, Pre-Treatment	VA, Post-Treatment	Complications/BT	Adjuvant Treatment
Peyman et al., 1984 [7]	34			Mean, 5.3 years (range, 1 to 10 years)	6% (2 pts died due to mets)	11 (32.3%)	14.7% (5 pts) incomplete resection		Postoperative VA ranged from 20/30 to light perception. Ten cases had VA > 20/200 (33%)	Most common operative complication: vitreous hemorrhage. Tractional RD occurred in 10 cases, 5 of which were inoperable, leading to phthisis bulbi and enucleation, cataract formation (9 pts), preretinal membranes, macular pucker, and chronic cystoid macular edema (4 pts).	No BT.
Foulds et al., 1986 [8]	140	Tumours up to 15 mm			9% (12 pts) 5-year survival 84%	Overall: 18% Recurrence 8.5%	14% 8.5% (12 pts) 1 orbital recurrence first cases		60% retained useful vision and 25% good vision	Not reported	No BT.
Shields et al., 1991 [9]	95	Mean 8.4 (range, 3.0 to 16.0)	Range, 2.0 to 12.0 (mean, 6.5)	Mean, 5.0 years; Median, 2.5 years (Range, 0.5 to 15.0 years)	5% mets (5 pts)	15 pts (16%)	15 pts (16%)		23 cases (24%) VA was ≥preoperative VA	VH 83%, intraretinal o subretinal hemorrhage 35% (most resolved spontaneously) RD: 28% Cataract: 34% RD surgery: 17%	No BT
Damato et al., 1996 [10]	332	13.2 (SD: 4.1)	7.4 (SD, 3.0)	Median: 36 m (Range, 42 days–20.9 years)	5-year mortality rates for pts with largest basal tumour diameters of <11 mm, 11–15 mm, and >15 mm: 3%, 20%, and 44%, respectively.						Adjuvant BT in 50 pts (15%)
Damato et al., 2006 [11]	344	Median 13 (range, 4 to 21.0)	Median 8 (range, 1.5 to 16.5)	Median: 13.0 years	16-year metastatic mortality was 20.2% if no risk factors *, 32.5% with one risk factor, and 46.7% with 2 risk factors.	8 y actuarial rates of eye preservation ranged from 57.1% (>1 ocular risk factor) to 81% for no risk factors ***	8-year actuarial rates of local tumour control were 75.2% in the absence of any risk factors **, 71.2% with one risk factor, and 47.1% with ≥1 risk factor	20/17–20/40 in 59.3%, 20/50–20/200 in 28.5%, counting fingers in 8.7%, and hand movements to light perception in 3.5%	8-year actuarial rates of conservation of vision of counting fingers or better were 63.9% if no risk factors *** were present, 60.1% with one risk factor, and 43.5% with more than one risk factor	RD tended to occur in the early postoperative period. The 2-year actuarial rate of RD was 16.6% (if the tumour height was <6 mm, increasing to 34.0% if the tumour was >9 mm).	Adjuvant BT: 129 pts (37.5%)
Bechrakis et al., 2010 [12]	150 included over 210	Median 14.5 (SD: 3.1) (range, 6.0–22.5)	Median 9.4 (SD: 2.0); (range, 2.0–14.6	Median 45.5 (SD 31.6) 3–142 months	5- and 10-year metastatic rates were 28% and 44%, respectively. Significant risk factors were extraocular spread, tumour thickness, and local tumour recurrence	9 pts (6%)	Residual tumour: 6%; Recurrence: 26 pts (17.3%); 5 and 10 year recurrence rates: 24% and 32%. Significant risk factors: older age, large basal diameter, lack of adjuvant ruthenium brachytherapy, and RD.		Not reported	No reported complications	Adjuvant BT in 136 pts (90.6%)

Abbreviations: BT, brachytherapy; VA, visual acuity; SD, standard deviation; mets, metastases; RD, retinal detachment; VH, vitreous haemorrhage; PLSU, partial lamellar sclerouvectomy; pts, patients. * Risk of metastatic death was categorised according to (1) basal tumour diameter > 15.4 mm; (2) tumour extension anterior to ora serrata; and (3) presence of epithelioid cells. ** Risk of local tumour recurrence was categorised in a similar fashion according to (1) basal tumour diameter > 15.4 mm; (2) posterior tumour extension to within 3.0 mm of optic disk or fovea; and (3) presence of epithelioid cells. *** Risk of adverse ocular outcomes was categorised according to the number of the following features: (1) basal diameter > 15.4 mm; (2) thickness > 8.4 mm; and (3) posterior extension to within 3.0 mm of the optic disk or fovea.

**Table 3:** Summary of case-control studies of partial lamellar sclerouvectomy for uveal melanoma.

Author	Treatment	Patients, n.	Diameter, mm	Thickness, mm	Follow-Up, mean	Survival	Enucleation	Recurrence	VA Pre-Treatment	VA Post-Treatment	BT
Foulds et al., 1987 (Matched for diameter) [13]	PLSU (Laser or BT adjuvant not all)	157	13.3 ± 4 mm maximum diameter			Overall 5 years 79% (26/33) <15 mm diameter 5-year mortality 11.6% and >16 mm 57%	22%	17% residual or recurrent 2% orbital recurrent		65% useful vision (between 6/6 and CF)	VH: 23% RD: 25% CT:15–61%
	Enucleation	241	13.3 ± 4 mm maximum diameter			5 years: 47% (89/188) 5-year mortality by diameter <15 mm: 30% >16 mm: 65%	100%	0		0	-
Augsburger et al., 1990 [14]	PLSU (No BT adjuvant)	30	9.8	5.7	3.0 y	3/30 KM 5 years: 85.2%			20/30	20/200 <20/200 at 5 years 66.8%	Significantly worse vision in PLSU
	BT Co60	30	10.7	5.8	6.0 y	7/30 KM 5 years: 81.8%			20/25	20/40 <20/200 at 5 years 44.4% (*p* = 0.0008)	No difference in mortality
Bechrakis et al., 2002 [15]	PLSU (BT Ruth-106 adj., not all)	85 (36 matched)	14.4 (±3.0)	9.5 (±2.1)	24.0 m (±16)	5.6% NO KM curves	11.1%	Persistent tumour: 13.9% Recurrence: 8.3%		≥20/200 retained in 61.1%	Cataract: 44.4% Additional VR surgery: 44.4% Peripheral cryo: 5.6% PRP:2.8% Neovascular glaucoma 5.6% Cyclocrio: 0%
	BT I-125	152 (80 matched)	14.6 (±2.4)	9.0 (±1.1)	33.0 m (±19)	11.1% NO KM curves	5.6%	Persisting tumour 5.6% Recurrence 5.6%		20/200 or better was retained in 5.6%	Cataract: 28% PRP: 66.6% Peripheral cryo: 11.1% Neovasc glaucoma 33.3% Cyclocryo: 5.6%
Kivela et al., 2003 [16] two centres only, one treatment in each centre (UK/ Finland retrospective study)	PLSU (BT Ruth106 adjuvant not all)	49	less than 12 mm for 13 pairs (26%), between 12 and 14 mm for 17 pairs (35%), and 14 mm or more for 19 pairs (39%).	Median 8.0 IQR (7.0–9.0)		18.3% (9 pts mets) 8 year all-cause and melanoma-specific survivals did not differ	Not reported Almost 32.6% (16 recurrences)	16 pts (32.6%) (14 pts, no adjuvant RT) 2 pts also developed an extrascleral recurrence	20/40 or better for 17 (35%) matched pairs, between 20/50 and 20/200 for 25 pairs (51%), and worse than 20/200 for 7 pairs (14%	The risk of losing 20/60 vision did not differ statistically after TSR and IBT The risk of losing 20/200 vision was significantly higher after IBT than after TSR	Higher risk of cataract, vitreous hemorrhage, maculopathy after IBT Rubeosis, neovascular glaucoma, and optic neuropathy developed only after IBT
	BT I-125	49	idem	Median 7.8 IQR (7.0–9.3)		22.4% (11 pts mets)	6.1% (3 pts)	6.1% (3 pts)			Significantly Higher risk of local recurrence after TSR The risk of retinal detachment did not differ between TSR and IBT
Puusaari et al., 2007 [11] two centres only, one treatment in each centre (UK/ Finland retrospective study)	PLSU (BT Ruth106 adjuvant not all)	33	Median 12.5 mm (range 9.8–16)	Median 11.0 mm (range, 8.0–14)	2.3 years	Not reported	24.2% (8 pts) 5-year incidence 28% (95% CI, 7–40)	27% (9 pts) 5-year incidence of local recurrence 41% (95% CI, 17–63) after TSR. 37% vs. 46% adjuvant irradiation vs. none.	33% (11 pts) in the TSR arm and 33% (18 pts) in the IBT arm had visual acuity better than 20/70	The mean visual acuity after TSR was 20/320 to 20/640 throughout the first 5 years and approximately one line better than the mean visual acuity after IBT, which varied between 20/640 and 20/1250 Cumulative incidence of loss of visual acuity 20/400 was 53% at one and 60% at 2 and 3 years	Adj. ruth BT. At 5 years, cataract in 91% after TSR and 78% after IBT. At 5 years, maculopathy in 63% after TSR and 43% after IBT. RD 5-year cumulative incidence 43%. decreased rapidly to <20% after TSR. Vitreous bleeding in 69% within 6 months of surgery but no new bleeding after this period. Glaucoma and optic neuropathy were rare after TSR
	BT I-125	54	14.0 mm (range, 7.3–16)	median, 10.6 mm; range, 8.2–13.3	5.3 years	Not reported	5 pts 5-year incidence 10% (95% CI 4–20)	3 pts 5-year incidence of local recurrence: 7% (95% CI, 2–17) after IBT		Cumulative incidence of loss of visual acuity 20/400 were 60% (95% CI, 44–73), 75% (95% CI, 59–86) and 91% (95% CI, 76–97)	Iris NV and glaucoma only in IBT optic neuropathy 5 years was 7% TSR and 58% in IBT RD exudative 5-year cumulative incidence 27% vitreous haemorrhage 5-year cumulative incidence of was 39% after IBT Cataract, maculopathy, RD, and vitreous haemorrhage were common after either treatment.
Caminal et al., 2016 [17] Resection without hypotensive anaesthesia	PLSU (adjvant BT in some but not all)	19	Median 14.7 (11.0–20.0)	Median 11 (4.0–12.0)	50.9 months (9.6–102.5	15.8% (3 mets) 10.5% (2 deaths) K-M at 5 years 79.1% without mets K-M DSS: 84% at 5 y	21.1% (5 pts) KM at 5 ears 70.9% maintain the eye	10.5% (3 pts) K-M at 5 years 82.5% free Recurrences in pts no adjuvant BT	20/200 (20/20.000–20/20)	20/34 (20/20.000–20/20) ≥20/200 or better: 53.3%	Better preservation of VA. Most common complications: rhegmatogenous RD (21.1%) and ocular hypertension (21.1%).
	I-125 BT	53	Median 15.5 (8.0–20.0)	Median 9 (6.0–11.0)	55.9 months (9.6–107.8)	26.4% (14 mets) 9.4% (5 deaths) K-M at 5 years: 74.2% without mets K-M at 5 years DSS: 93.2%	9.4% (4 pts) KM at 5 y 89.8%	5.7% (2 pts) K-M at 5 years 94.1% free	20/40 (20/20.000–20/20)	20/20.000 (20/200.000–20/25) ≥20/200: 31.2%	Most common complications: radiation-induced retinopathy (45.3%), neovascular glaucoma (28.3%) and macular oedema. (24.5%)

Abbreviations: PLSU: partial lamellar sclerouvectomy; CI, confidence interval; DSS, disease-specific survival; PRP: Panretinal photocoagulation; BT: Brachytherapy; VR surgery: vitreoretinal surgery; KM: Kaplan-Meier; CF: counting fingers; RD: retinal detachment; mets, metastasis; IBT, iodine brachytherapy; TSR, transscleral resection.

**Table 4:** Summary of studies on endoresection for uveal melanoma.

Authors	Patients, n	Diameter, mm	Thickness, mm	Follow-Up, mean	Survival	Enucleation	Recurrence	VA Pretreatment	VA Posttreatment	Complications
Kertes et al., 1998 [18] Case series retrospective No adjuvant BT	32	8.0 (range 3–18)	5.3 (flat to 12.0)	40	9.4% (3 pts) died	9.4% (3 pts)	3.1% (1 pts)		31.2% had VA ≥ 6/60; 56.3% between 6/120 and light perception, 12.5% NLP	VH (37.5%); cataract (25%); RD (9.3%); glaucoma (9.3%).
Damato et al., 1998 [19] Retrospective case series Adjuvant BT selected pts	52	8.2 (range 4.0–14.0)	3.9 (1.5–10.1)	20	0	10%	0		90% eye retention	RD (16), cataract (25), ocular hypertension (14), phthisis (one), epiretinal membrane (one), VH (two), possible local tumour recurrence (eight), and endophthalmitis (one)
Garcia-Arumi et al., 2001 [20] Retrospective case series Adjuvant BT selected pts	25	12.1 (range, 8.9–14.8)	10.6 (range, 9.1–12.8)	31 (12–72)	0	0	0	Mean, 20/60 (range, 20/400 to 20/20)	Hand motions to 20/30 (mean, 20/100)	Hemorrhage at the scleral bed (100%), cataract (40%), ocular hypertension (32%), RD (16%), macular traction (16%), epiretinal macular proliferation (8%), branch vein occlusion (4%), and submacular hemorrhage (4%).
Bechrakis et al., 2006 [21] Retrospective case series Adjunctive Proton radiotherapy	58	15.6 (11.1–21.6)	Median: 8.8 (range, 7.0–14.5)	Median 18 m (3.0–48)	4% (2 mets)	KM at 2 years was 8.4%	2% (1 recurrence at 40 m)	20/40 (range, 20/400–20/20)	Median: 20/200	KM at 2 years: Cataract: 46.6%; RD 32.1%; Macular hole 4.0%; Secondary glaucoma 2.6%; Phthisis 2.1%; Macular pucker 1.8%; Radiation retinopathy: 27.5%; radiation optic neuropathy: 29.4%
Karkhaneh et al., 2007 [22] Retrospective case series No adjuvant BT	20	11.67 (range, 8.0–15.7)	8.51 (range, 5.5–11)	89.55 months (24–132)	5% (1 pt)	15% (3 pts)	10% (2 pts)	Mean, 20/100 (hand motions to 20/30)	No light perception to 20/30	Cataract: 25%: RD: 15%; Bullous keratopathy: 10%
Garcia-Arumi et al., 2008 [23] Retrospective case series BT all pts	38	9.9 (5–15)	10.1(7.7–13.5)	Mean 70.63 (23 to 129)	13% (3/23) 90.9% at 5 years (K-M)	7.9% (3 pts)	5.8% (2 pts)	20/60 (‘‘hand-movements’’ to 20/20)	Mean 20/300 (‘‘no light perception’’ to 20/30)	Hemorrhage at the scleral bed: 100%; ocular hypertension: 31.5%; RD: 26%; epiretinal macular proliferation: 11%; post-radiation retinopathy: 5.2%; and subretinal neovascularisation: 5.2% at 2 years
Konstantinidis et al., 2014 [24] NO adjuvant BT	71	9.5 (4.8–14.5)	4.4 (0.9–11.1)	49.2 m (range, 24 to 195.6 m)	7% (5/71) 9% at 5 years and 10 years	4%	3% (2 pts) 3.7% at 10 years KM		Better than 6/12 in 9 (13%) and better than 6/30 in 21 (31%)	Cataract (94%), transient ocular hypertension (10%), epiretinal membrane (13%) and moderate haemorrhage (3%); RD (22%).
Garcia-Arumi J. et al., 2015 [25] 20 pat endoresection alone 21 pts BT	41	9.9 (range, 5–15)	9.8 (range, 7.7–13.5)	102.5 (20–180)	7.3% (3 pts) KM at 10 years 97.6%	12..2%	5 pts (12.2%) in the group endo alone	20/100 (range, hand movement–20/20)	No light perception to 20/20, with mean 20/1625	RD 28.9%; Phthisis bulbi 12.2%; PRP 9.8%; ERM 12.2%; CNVM 4.9%; Subretinal fibrosis 2.4%; Ocular hypertension 34.1%; VH 2.4%; CME 2.4%; corneal decompensation 2.4%
Biewald E et al., 2017 [26] Prior treatment with gamma and postop BT	200	12 (range, 6.3–20)	9.4 (range 6–14.8)	32.3 months	15.5% (31 /200)	11% (22/200)	5% (10 pts)		13.4% the VA of 20/50 or better and 20/400 to 20/50 in 33.6%	Cataract surgery in 70%; pars plana vitrectomy revision surgery in 1.5%; use of an adjuvant ruthenium-106 plaque did not lower recurrence
Vidoris et al., 2017 [27] No adjuvant BT	14	15.98 ± 4.76 (range: 4.3–23)	6.05 ± 1.94 (range: 3.3–9.7)	Mean 54.5 (range: 12–60 m)	7.1% (1 pt)	0	0		Between 20/60 and 20/200 (78.5%)	Macular bleeding (7.1%); elevated IOP (7.1%); RD (14.2%)
Susskind et al., 2017 Adjuvant BT [28]	35	Mean 13.0 (8..4–17.6)	Mean: 7.33 (range, 4.06–12.2)	Mean: 65.7 (12 and 154)	20% (7 pts) 92.0% 5 years and 57.9% 10 years KM	22.9% (8 pts) 77.3% 5 years and 70.8% 10 years KM	22.9% (8 pts) (22.7% at 5 years and 29.2% at 10 years (KM)	Median 0.2 (2.3–0) LogMar	Median 2.0 (4.0–0.4) LogMar	Radiation retinopathy: 11.4%; CNV: 8.6%); pale optic disc (opticopathy): 31.4%; corneal decompensation: 8.6%; epiretinal membrane: 5.7%; RD PVR: 11.4%; macular hole: 2.8%; persistent corneal erosion: 2.8%; corneal band keratopathy: 5.7%; persistent hypotony: 5.7%.

Abbreviations: CNV, choroidal neovascularization; IOP, intraocular pressure; RD, retinal detachment. CME: cystoid macular edema; CNVM: Choroidal neovascular membrane; ERM: epiretinal membrane; PRP: postradiation retinopathy; VH: vitreous hemorrhage; pts, patients.

**Table 5:** Summary of case-control studies of endoresection for uveal melanoma.

Author	Treatment	Pts, n	Diameter, mm	Thickness	Follow-Up, mean	Survival	Enucleation	Recurrence	VA, Pre-Treatment	VA, Post-Treatment	Complications
Caminal et al., 2013 [29]	Endoresection (no BT adjuvant)	27	11 (IQR 2.9)	7.69 (IQR 2.27)	59.37 (12–138)	3.7% (1/27) metastasis 3.7% specific death 100% at 5 years (K-M) cause-specific survival	11.1% (3 pts) 87.8% at 5 years (K-M)	7.4% (2 pts) 92.4% at 5 years (K-M)	0.2	22.2% 20/200 or better KM probability of maintaining a VA equal or superior to 20/200 at 5 years was 59.9%	
	BT	54	12.5 (IQR 2.0)	6.95 (IQR1.8)	70.52 (12–148)	20.4% (11 pts) 18.5% specific death 84.2% at 5 years (K-M) cause-specific survival	13% (7 pts) 85.7% at 5 years (K-M)	1.8% (1 pts) 96.6% at 5 years (K-M)		35.2% 20/200 or better KM probability of maintaining a VA equal or superior to 20/200 at 5 years was 66.4% group and 59.9% in the endoresection group	
Rice et al., 2014 [30]	Endoresection (BT adjuvant)	22	11.2(3.4–16.0)	7.3 mm (3.0–10.0)	62.4 (7.9–121.8)	18.2% metastasis 20.4% died	4.6% (1 pts)	18.2% (4 pts)	Snellen ±6/24	±6/50 6/18 or better in 41% of endoresection group and 35% of the BT group retained vision of 6/18 or better at the end of follow-up. Eight out of 22 (36%) endoresection and 68/142 (48%) BT had 2/60 or worse at the last visit	
	BT	148	10.3 mm (range 4.5–16.0)	4.9 mm (range 2.5–10.0	55.4 (6.6–175.8)	14.2%	10.8%	Local recurrence rate higher in endoresection group (18.2% vs. 14.9%, *p* = 0.75) but not significant.			

Abbreviations: IQR, interquartile range; pts, patients; BT, brachytherapy; CME: cystoid macular edema; CNVM: Choroidal neovascular membrane; ERM: epiretinal membrane; PRP: postradiation retinopathy; VH: vitreous hemorrhage; pts, patients.

## Data Availability

Not applicable.

## References

[1] Singh M., Durairaj P., Yeung J. (2018). Uveal Melanoma: A Review of the Literature. Oncol. Ther..

[2] Shields J.A., Shields C.L. (2015). Management of Posterior Uveal Melanoma: Past, Present, and Future. Ophthalmology.

[3] Kapoor A., Beniwal V., Beniwal S., Mathur H., Kumar H.S. (2016). Management of uveal tract melanoma: A comprehensive review. J. Egypt. Natl. Cancer Inst..

[4] Gunduz K., Bechrakis N. (2010). Exoresection and endoresection for uveal melanoma. Middle East Afr. J. Ophthalmol..

[5] Damato B., Groenewald C. (2007). Uveal Malignant Melanoma: Management Options—Resection Techniques. Clinical Ophthalmic Oncology.

[6] Rospond-Kubiak I., Damato B. (2014). The surgical approach to the management of anterior uveal melanomas. Eye.

[7] Peyman G.A., Juarez C.P., Diamond J.G., Raichand M. (1984). Ten years experience with eye wall resection for uveal malignant melanomas. Ophthalmology.

[8] Foulds W.S., Damato B.E. (1986). Alternatives to Enucleation in the Management of Choroidal Melanoma. Aust. N. Z. J. Ophthalmol..

[9] Shields J.A., Shields C.L., Shah P., Sivalingam V. (1991). Partial lamellar sclerouvectomy for ciliary body and choroidal tumors. Ophthalmology.

[10] Damato B.E., Paul J., Foulds W.S. (1996). Risk factors for metastatic uveal melanoma after trans-scleral local resection. Br. J. Ophthalmol..

[11] Puusaari I., Damato B., Kivelä T. (2006). Transscleral local resection versus iodine brachytherapy for uveal melanomas that are large because of tumour height. Graefe’s Arch. Clin. Exp. Ophthalmol..

[12] Bechrakis N.E., Petousis V., Willerding G., Krause L., Wachtlin J., Stroux A., Foerster M.H. (2010). Ten-year results of transscleral resection of large uveal melanomas: Local tumour control and metastatic rate. Br. J. Ophthalmol..

[13] Foulds W.S., Damato B.E., Burton R.L. (1987). Local resection versus enucleation in the management of choroidal melanoma. Eye.

[14] Augsburger J.J., Lauritzen K., Gamel J.W., DeBrakeleer D.J., Lowry J.C., Eisenman R. (1990). Matched group study of surgical resection versus cobalt-60 plaque radiotherapy for primary choroidal or ciliary body melanoma. Ophthalmic Surg..

[15] Bechrakis N.E., Bornfeld N., Zöller I., Foerster M.H. (2002). Iodine 125 plaque brachytherapy versus transscleral tumor resection in the treatment of large uveal melanomas. Ophthalmology.

[16] Kivelä T., Puusaari I., Damato B. (2003). Transscleral resection versus iodine brachytherapy for choroidal malignant melanomas 6 millimeters or more in thickness: A matched case-control study. Ophthalmology.

[17] Caminal J.M., Padrón-Pérez N., Arias L., Masuet-Aumatell C., Gutiérrez C., Piulats J.M., Pera J., Català J., Rubio M.J., Arruga J. (2016). Transscleral resection without hypotensive anaesthesia vs. iodine-125 plaque brachytherapy in the treatment of choroidal melanoma. Eye.

[18] Kertes P.J., Johnson J.C., Peyman G.A. (1998). Internal resection of posterior uveal melanomas. Br. J. Ophthalmol..

[19] Damato B., Groenewald C., McGalliard J., Wong D. (1998). Endoresection of choroidal melanoma. Br. J. Ophthalmol..

[20] García-Arumí J., Sararols L., Martinez V., Corcostegui B. (2001). Vitreoretinal surgery and endoresection in high posterior choroidal melanomas. Retina.

[21] Bechrakis N.E., Foerster M.H. (2006). Neoadjuvant proton beam radiotherapy combined with subsequent endoresection of choroidal melanomas. Int. Ophthalmol. Clin..

[22] Karkhaneh R., Chams H., Amoli F.A., Riazi-Esfahani M., Ahmadabadi M.N., Mansouri M.R., Nouri K., Karkhaneh A. (2007). Long-term surgical outcome of posterior choroidal melanoma treated by endoresection. Retina.

[23] García-Arumí J., Zapata M.A., Balaguer O., Fonollosa A., Boixadera A., Martinez-Castillo V. (2008). Endoresection in high posterior choroidal melanomas: Long-term outcome. Br. J. Ophthalmol..

[24] Konstantinidis L., Groenewald C., Coupland S.E., Damato B. (2014). Long-term outcome of primary endoresection of choroidal melanoma. Br. J. Ophthalmol..

[25] Garcia-arumi J., Leila M., Zapata M.A., Velázquez D., Dinares-fernandez M.C., Tresserra F. (2015). Endoresection technique with /without brachytherapy for management of high posterior choroidal melanoma: Extended Follow-up Results. Retina.

[26] Biewald E., Lautner H., Gök M., Horstmann G.A., Sauerwein W., Flühs D., Bornfeld N. (2017). Endoresection of large uveal melanomas: Clinical results in a consecutive series of 200 cases. Br. J. Ophthalmol..

[27] Vidoris A.A.C., Maia A., Lowen M., Morales M., Isenberg J., Fernandes B.F., Belfort R.N. (2017). Outcomes of primary endoresection for choroidal melanoma. Int. J. Retin. Vitr..

[28] Süsskind D., Dürr C., Paulsen F., Kaulich T., Bartz-Schmidt K.U. (2017). Endoresection with adjuvant ruthenium brachytherapy for selected uveal melanoma patients—The Tuebingen experience. Acta Ophthalmol..

[29] Caminal J.M., Mejia K., Masuet-Aumatell C., Arias L., Piulats J.M., Gutierrez C., Pera J., Catala J., Rubio M., Arruga J. (2013). Endoresection versus iodine-125 plaque brachytherapy for the treatment of choroidal melanoma. Am. J. Ophthalmol..

[30] Rice J.C., Stannard C., Cook C., Lecuona K., Myer L., Scholtz R.P. (2014). Brachytherapy and endoresection for choroidal melanoma: A cohort study. Br. J. Ophthalmol..

[31] Zirm E. (1911). Über endobulbare operationen. Arch Augenheilkd.

[32] Stallard H.B. (1966). Partial choroidectomy. Br. J. Ophthalmol..

[33] Foulds W.S. (1973). The local excision of choroidal melanomata. Trans. Ophthalmol. Soc. U. K..

[34] Peyman G.A., Axelrod A.J., Graham R.O. (1974). Full-Thickness Eye Wall Resection: An Experimental Approach for Treatment of Choroidal Melanoma: Evaluation of Cryotherapy, Diathermy, and Photocoagulation. Arch. Ophthalmol..

[35] Shields J.A., Augsburger J.J., Stefanyszyn M.A., Connor R.W. (1984). Sclerochorioretinal resection for choroidal melanoma. A clinicopathologic correlation of a postmortem eye. Ophthalmology.

[36] Shields J.A., Shields C.L. (1988). Surgical approach to lamellar sclerouvectomy for posterior uveal melanomas: The 1986 Schoenberg lecture. Ophthalmic Surg..

[37] Damato B. (2006). The role of eyewall resection in uveal melanoma management. Int. Ophthalmol. Clin..

[38] Peyman G.A., Apple D.J. (1974). Local Excision of Choroidal Malignant Melanoma: Full-Thickness Eye Wall Resection. Arch. Ophthalmol..

[39] Meyer-Schwickerath G. (1974). Excision of malignant melanoma of the choroid. Mod. Probl. Ophthalmol..

[40] Damato B., Foulds W.S. (1996). Indications for trans-scleral local resection of uveal melanoma. Br. J. Ophthalmol..

[41] Char D.H., Miller T., Crawford J.B. (2001). Uveal tumour resection. Br. J. Ophthalmol..

[42] Kim J.W., Damato B.E., Hiscott P. (2002). Noncontiguous tumor recurrence of posterior uveal melanoma after transscleral local resection. Arch. Ophthalmol..

[43] Damato B.E., Paul J., Foulds W.S. (1996). Risk factors for residual and recurrent uveal melanoma after trans-scleral local resection. Br. J. Ophthalmol..

[44] Damato B. (1997). Adjunctive plaque radiotherapy after local resection of uveal melanoma. Front. Radiat. Ther. Oncol..

[45] Damato B., Groenewald C.P., McGalliard J.N., Wong D. (2002). Rhegmatogenous retinal detachment after transscleral local resection of choroidal melanoma. Ophthalmology.

[46] Damato B.E., Paul J., Foulds W.S. (1993). Predictive factors of visual outcome after local resection of choroidal melanoma. Br. J. Ophthalmol..

[47] Peyman G.A., Barrada A. (1984). Retinochoroidectomy ab interno. Ophthalmic Surg..

[48] Peyman G.A., Cohen S.B. (1986). Ab interno resection of uveal melanoma. Int. Ophthalmol..

[49] Robertson D.M.M. (2001). Melanoma Endoresection: A Perspective. Retin. J. Retin. Vitr. Dis..

[50] Garcia-arumi J., Distefano L.N., Quijano C. (2015). Endoresection of a high equatorial choroidal melanoma. Retina.

[51] Ledowski T., Kiese F., Jeglin S., Scholz J. (2005). Possible air embolism during eye surgery. Anesth. Analg..

[52] Rice J.C., Liebenberg L., Scholtz R.P., Torr G. (2014). Fatal air embolism during endoresection of choroidal melanoma. Retin. Cases Brief Rep..

[53] Morris R.E., Sapp M.R., Oltmanns M.H., Kuhn F. (2014). Presumed air by vitrectomy embolisation (PAVE) a potentially fatal syndrome. Br. J. Ophthalmol..

[54] Huang Y., Wei W.-B. (2016). Acute pulmonary embolism caused by local resection of choroidal melanoma. Chin. Med. J..

[55] Gayer S., Palte H.D., Albini T.A., Flynn H.W., Martinez-Ruiz R., Salas N., McClellan A.J., Relhan N., Parel J.-M. (2017). In-Vivo Porcine Model of Venous Air Embolism During Pars Plana Vitrectomy. Am. J. Ophthalmol..

[56] Suesskind D., Ulmer A., Schiebel U., Fierlbeck G., Spitzer B., Spitzer M.S., Bartz-Schmidt K.U., Grisanti S. (2011). Circulating melanoma cells in peripheral blood of patients with uveal melanoma before and after different therapies and association with prognostic parameters: A pilot study. Acta Ophthalmol..

[57] Diener-West M., Earle J.D., Fine S.L., Hawkins B.S., Moy C.S., Reynolds S.M., Schachat A.P., Straatsma B.R., Collaborative Ocular Melanoma Study Group (2001). The COMS randomized trial of iodine 125 brachytherapy for choroidal melanoma, III: Initial mortality findings. COMS Report No. 18. Arch Ophthalmol..

[58] Damato B., Wong D., Green F.D., Mackenzie J.M. (2001). Intrascleral recurrence of uveal melanoma after transretinal “endoresection”. Br. J. Ophthalmol..

[59] Mittica N., Vemuganti G.K., Duffy M., Torczynski E., Edward D.P. (2003). Late Orbital Recurrence of a Choroidal Melanoma Following Internal Resection. Surv. Ophthalmol..

[60] Ruest P., Aroichane M., Cordahi G., Bureau N. (2007). Possible venous air embolism during open eye surgery in a child. Can. J. Anaesth..

[61] Dermigny F., Daelman F., Guinot P.-G., Hubert V., Jezraoui P., Thomas F., Milazzo S., Dupont H. (2008). Fatal air embolism during open eye surgery. Ann. Fr. Anesth. Reanim..

